# Leaf Extracts of *Moringa oleifera* Cultivated in Baghdad: Characterization and Antimicrobial Potential against Endodontic Pathogens

**DOI:** 10.1155/2024/6658164

**Published:** 2024-02-28

**Authors:** Nada E. Shafiq, Anas F. Mahdee, Zainab Y. Mohammed Hasan

**Affiliations:** ^1^Restorative and Aesthetic Dentistry Department, College of Dentistry, University of Baghdad, Baghdad, Iraq; ^2^Biotechnology Research Center Al-Nahrain University, Baghdad, Iraq

## Abstract

The use of medicinal plant preparations to clean and disinfect root canal infection is gaining popularity. The aim of this study was to evaluate the bioactive composition of leaf extracts of *Moringa oleifera* plants cultivated in Iraq (specifically Baghdad) and their antimicrobial activity against selected root canal pathogens for potential application in endodontic treatment. *Materials and Methods*. *Moringa* leaf extracts were prepared either through cold maceration or warm digestion techniques to perform an ethanolic or aqueous extraction, respectively. Phytochemical detection was performed before thin layer chromatography (TLC) and high-performance liquid chromatography (HPLC) to measure flavonoids and phenolic compounds within both extracts. Then, their antimicrobial activities were investigated against *Streptococcus mutans*, *Enterococcus faecalis*, and *Candida albicans* through minimal inhibitory concentration (MIC), minimal bactericidal concentration (MBC), and agar well diffusion assay in comparison to NaOCl and Ca(OH)_2_. *Results*. Phytochemical screening showed several active ingredients but with higher expression of flavonoids and phenolic compounds. Also, different types of these compounds were detected through TLC and quantified by HPLC. MIC values for ethanolic extract against *Streptococcus mutans*, *Enterococcus faecalis*, and *Candida albicans* were 60, 65, and 55, respectively, while for aqueous extract, MIC values were 70, 80, and 50, respectively. Aqueous extract showed a higher inhibition zone than ethanolic extract for both *Streptococcus mutans* and *Enterococcus faecalis* with a statistically significant difference (*p* ≤ 0.001) for all tested materials except with NaOCl and Ca(OH)_2_ in *Streptococcus mutans* and *Enterococcus faecalis*, respectively. The ethanolic extract showed a higher inhibition zone against *Candida albicans*, with a statistically significant difference (*p* ≤ 0.001) for all tested materials. *Conclusion*. Ethanolic and aqueous extracts of *Moringa oleifera* leaves cultivated in Baghdad contain considerable quantities of phytochemicals, especially flavonoid and phenolic compounds, and demonstrated antimicrobial activities against selected endodontic pathogens. Therefore, *Moringa* leaf extracts could be suggested as an alternative antimicrobial material in endodontic treatment.

## 1. Introduction

Herbal materials are increasingly valued in dental and medical practice due to their antimicrobial, antioxidant, anti-inflammatory, and biocompatibility properties [1]. One of these medicinal plants is *Moringa oleifera* (*M. oleifera*) that belongs to the Moringaceae family and is commonly called horseradish or drumstick tree [2]. This plant possesses significant nutritional and medicinal properties which make it a good source of glucosinolates, flavonoids and phenolic acids, carotenoids, tocopherols, polyunsaturated fatty acids, minerals, and folate [3]. *M. oleifera* is also claimed to possess antibacterial, antifungal, anti-inflammatory, antioxidant, antiasthmatic, antiulcer, antidiabetic, antitumor, antipyretic, antiepileptic, diuretic, antihypertensive, cholesterol lowering, and hepatoprotective properties [4]. The biochemistry of *M. oleifera* varies according to the cultivation region, and such variation in the chemical composition can result in divergent effectiveness on health problems and safety related to its intake [5].

In endodontic treatment, the dominant factors for pulpal and periapical inflammation are bacteria and their byproducts [6]. Thus, elimination of them from the contaminated root canal system by both mechanical and chemical means is essential to achieve successful results [7]. Lately, the use of herbal medicines in dental treatment has increased, giving the advantages of availability, lesser toxicity, and cost effectiveness [8]. Herbal agents have been used in dentistry as anti-inflammatory agents, antimicrobial plaque agents, antioxidants, analgesics, endodontic irrigants, and medicaments [9, 10]. With the high nutritional and medicinal value of *M. oleifera*, scientific research has been directed toward this medicinal herb with antioxidant, anti-inflammatory, and antimicrobial activities as a possible source of antimicrobial function in endodontic treatment [11]. Leaf and seed extracts demonstrated antibacterial activity against *E. faecalis in vitro* and in the root canal *ex vivo* [12–14]. Thus, the present study focused on the bioactive composition and biological functions of leaf extracts from *M. oleifera* plants cultivated in Iraq (specifically Baghdad) to explore their antimicrobial action against selected root canal pathogens.

## 2. Materials and Methods

### 2.1. Plant Collection and Classification

Fresh *M. oleifera* plant leaves were collected from the plant research garden of the Department of Biology/College of Science, University of Baghdad. The plant was identified and authenticated properly at the Herbarium of the College of Science, University of Baghdad. The leaves were left to dry in the shade at room temperature [15] and then ground into a fine powder using an electric blender.

### 2.2. Plant Extracts Preparation

Two types of plant extracts were prepared. Ethanolic extract was prepared by the cold maceration method as described by Ibrahim and Kebede [16]. Using this technique, 100 g of dried leaf powder was added gradually to 1000 mL of 80% ethanol (PanReac AppliChem, Spain) in a glass beaker under stirring and stored for 72 hours. The extract was filtered via Whatman filter paper No. 1 and dried by using a rotatory evaporator (Heiodolph, Germany) at 60°C to obtain the dried extract (residue). The residue was stored at 4°C until use. The aqueous extract was prepared by the digestion method described by Abubakar and Haque [17] as follows: 50 g of the dried leaf powder was added to 1500 mL of distilled water and placed on a hotplate magnetic stirrer (Rlabinco, the Netherlands) at 60°C for one and a half hours before filtering through Whatman filter paper No. 1. The filtrate was freeze-dried with a lyophilizer apparatus (Christ ALPHA 2–4 LD plus, Martin Christ Gefriertrocknungsanlagen GmbH).

The percentage yields of both extracts were calculated using the following formula [18]:

(1) $$\begin{array}{c} \text{Percentage }\left(\%\right)\text{ yield}=\frac{\text{weight }\left(g\right)\text{ of the concentrated extract }}{\text{weight }\left(g\right)\text{ of the ground Moringa leaves}}\;x\;100. \end{array}$$

Also, pH was measured using a pH meter (WTW, Germany) for a solution of 5 mg of each residue in 10 mL distilled water.

### 2.3. Preliminary Phytochemical Detection of the Ethanolic and Aqueous Extracts

The following tests were then used to detect the presence of polyphenols, flavonoids, tannins, alkaloids, saponins, and polysaccharides.

#### 2.3.1. Test for Flavonoids

This was performed according to Abubakar and Haque [17] and Shri Chengama Raju and Wing Kei [19]. A few drops of sodium hydroxide solution were added to a glass tube containing 1 mL of extract, which turned the solution to an intense bright yellow color. Then, a few drops of dilute acid were added. If this turns the solution colorless, it indicates the presence of flavonoids.

#### 2.3.2. Test for Alkaloids

This was performed according to Abubakar and Haque [17]. The potassium bismuth iodide solution (Dragendorff's reagent) was freshly prepared using 60 mg of bismuth subnitrate Bi(NO_3_)_3_.H_2_O that was dissolved in 0.2 ml HCL (solution A) and 600 mg potassium iodide KI in 1 ml distilled water (solution B). Then, solutions A and B were mixed together. Following that, 1 mL of this reagent was added to the extract in a glass tube. The formation of an orange brown precipitate indicates the presence of alkaloids.

#### 2.3.3. Test for Tannins

A few drops of 1% lead acetate were added to 1 mL of the extract in a glass tube leading to the appearance of white or gelatinous precipitate. This indicates the presence of tannins [20, 21].

#### 2.3.4. Test for Glycosides

1 mL of extract solution was placed in a glass tube and treated with a few drops of Benedict's reagent (alkaline solution containing cupric citrate complex) before boiling in a water bath for 5 minutes and then cooling. The formation of a reddish brown precipitate means that a reducing sugar is present [22].

#### 2.3.5. Test for Saponins

Froth tests as described by Muttalib and Naqishbandi [23] and Pandey and Tripathi [22] were performed by continuous agitation of a glass tube containing 5 mL of extract for about 15 min. The formation of foam is indicative of the presence of saponins.

#### 2.3.6. Test for Polyphenols

As described by Abubakar and Haque [17], 1 mL solution of extract was mixed with 5% ferric chloride solution. The formation of a brown precipitate indicates the presence of polyphenols.

### 2.4. Thin Layer Chromatography (TLC) (Qualitative Assessment)

Reagents used were chloroform (Gainland Chemical Company, UK), ethanol (PanReac AppliChem, Spain), ethyl acetate (Gainland chemical Company, UK), formic acid (Thomas Baker, India), glacial acetic acid (Scharlau, Spain), and methanol (Alpha Chemika, India).

This test was used to separate the components of the extracts. The following mobile phases were tested: glacial acetic acid : chloroform : formic acid 0.7 : 8.8 : 0.5 [24], chloroform : methanol : water 7 : 3 : 1 [25], chloroform : methanol : ethanol 1 : 1 : 1 [26], chloroform : glacial acetic acid : methanol 4 : 5 : 1 [26], chloroform : methanol 9 : 1, and ethyl acetate : formic acid 9 : 1 [21]. The solvent selected for the separation of phenolic compounds and flavonoids was chloroform : glacial acetic acid : methanol (4 : 5 : 1), as it provides the best separation of the active compounds.

A thin layer chromatography, aluminum-backed TLC (SiliCycle, Canada), was activated at 100°C for 30 minutes in an oven and cooled at room temperature before use. All standard solutions for flavonoids and phenolic compounds were prepared at a concentration of 1 mg/mL in absolute methanol, including caffeic acids CA, rutin R, catechin CAT, epicatechin Ep, chlorogenic acid Ch, hydroquinone H, gallic acid G, cinnamic acid Cin, kaempferol K, quercetin Q, quarcitrin Qa, luteolin L, paracumaric acid PC, pyrogallol P, and apigenin A. One spot from standard solutions and one from each sample (ethanolic and aqueous extracts in concentration of 5 mg/mL) were placed on a TLC plate using capillary tubes. The plate was placed in a TLC jar containing the selected solvent. At the end of the solvent development, the developed TLC plates were air-dried and observed under ultraviolet light, UVT-260D Dual UV Transilluminator (Optima, Japan) at both 254 nm and 366 nm. Calculation of the retardation factor *R*_**f**_ value was done according to the following formula [27]:

(2) $$\begin{array}{c} R_{f}=\frac{\text{distance traveled by the compound}}{\text{distance traveled by the solvent}}. \end{array}$$

### 2.5. High-Performance Liquid Chromatography (HPLC) (Quantitative and Qualitative Assessment)

Both ethanolic and aqueous extracts in 10 mg/mL methanol were prepared for the detection of flavonoids and phenolic compounds and analyzed using an HPLC system (Shimadzu, Japan). The conditions for detection were as shown in Table 1 for phenolic compounds and Table 2 for flavonoids.

The concentration of the detected compounds was measured according to the following formula [28]:

(3) $$\begin{array}{c} \text{Concentration of unknown}=\frac{\text{area of unknown }}{\text{area of known}}X\text{ concentration of known}. \end{array}$$

### 2.6. Antimicrobial Testing

Microbial cultures used were microbial strains of *Streptococcus mutans* (OP198206.1), *Enterococcus faecalis* (OM250466.1), and *Candida albicans* (OP683214.1) that were previously isolated and identified through real-time PCR. These microbes were selected because of their significance in endodontic infections [29]. The microbial strains (*Streptococcus mutans*, *Enterococcus faecalis*, and *Candida albicans*) were cultured in Muller Hinton broth (Oxoid Ltd., United Kingdom) and incubated overnight in an Electro-Thermal Constant-Temperature Incubator (Laboao, China) at 37°C and then diluted with 1 : 10 dilution factor with Muller Hinton broth (Oxoid Ltd., United Kingdom). The turbidity of the suspensions was adjusted to obtain 0.5 McFarland standard, which estimates a concentration of (1 × 10^8^ CFU/ml). These microbial suspensions were used in the following test.

#### 2.6.1. Minimum Inhibitory Concentration (MIC)

The MIC is defined as the lowest concentration of the antimicrobial agent which has the ability for complete inhibition of microorganism growth in tubes or microdilution wells as noticed by the unassisted eye [30].

The MIC test for the ethanolic and aqueous extracts was done using a 96-well plate microdilution method (resazurin microtiter assay (REMA) plate) [31, 32], as follows.

Muller Hinton broth was prepared aseptically. Muller Hinton broth was used to prepare seven concentrations of each extract. These concentrations for the ethanolic extract were 75 mg/mL, 70 mg/mL, 65 mg/mL, 60 mg/mL, 55 mg/mL, 50 mg/mL, and 45 mg/mL, while for the aqueous extract, they were 90 mg/mL, 80 mg/mL, 70 mg/mL, 60 mg/mL, 50 mg/mL 40 mg/mL, and 30 mg/mL. Briefly, in each row of a flat-bottom 96-well plate, about 150 *μ*L of each extract's dilution was dispended into 11 wells (10 replications for each concentration) and one last well was used as a blank for the color changing. While, 150 *μ*L of Muller Hinton broth was added to each well of the last column of the plate to serve as the positive control (which would later be inoculated with microbial isolation). In each well of the last row of the plate, 200 *μ*L of Muller Hinton broth with no tested materials was added to serve as the negative control. Then, 50 *μ*L of the previously prepared microbial isolate suspension was added to each well except for the blank and the negative control wells. After overnight incubation at 37°C, 30 *μ*L of 0.015% solution of resazurin (HiMedia, India) was added to each well and they were incubated again for four hours.

A color change was assessed visually in the prepared plates. Any change in color from blue to pink-orange was recorded as positive, indicating microbial growth, while no color change (blue resazurin color remained unchanged) indicated that no microbial growth had occurred. The blank wells were used only to check if there was any reaction between the extracts and resazurin pigments. The MIC showed the lowest concentration of the tested extract at which no color change of the medium appeared and no microbial growth was indicated [33].

#### 2.6.2. The Minimum Bactericidal Concentration (MBC)

It is the lowest concentration of the antibacterial agents which can entirely kill the bacteria [34]. It was determined to use the same method as Jang et al. [35] and Prastiyanto et al. [36], and the procedure was done by taking 10 *μ*L from wells of the lowest 3 MIC values and one sample from the concentration below the MIC value from each plate that was used to determine the MIC against the tested microorganisms for both extracts. Then, these suspensions were spread evenly on blood agar base plates (TM MEDIA, India). The plates were incubated for 24 hours in order to detect any microbial colony growth. When no microbial colony growth occurred from directly plated contents of these selected wells, this value was recorded as the MBC value [32].

#### 2.6.3. Microbial Sensitivity Test

The aim is to determine the diameter of the inhibitory concentration of the ethanolic and aqueous extracts using the agar well diffusion assay as described by Prastiyanto et al. [36, 37].

Five different plates of Mueller Hinton agar were inoculated with the previously prepared microbial isolates suspension. In each plate, 4 holes of 8 mm diameter were punched aseptically with a sterile cork borer. Two of these holes received 100 *μ*L of the MIC concentration for either ethanolic or aqueous extracts. The other two holes received either calcium hydroxide paste material (Meta Biomed, Korea) or 3% sodium hypochlorite solution (CLORMIX, Iraq) as comparative materials. Then, the plates were incubated overnight at 37°C, and the diameter of the inhibition zones was measured.

The data were analyzed using analysis of variance ANOVA and post-hoc Bonferroni multiple comparisons at the 0.05 level.

## 3. Results

### 3.1. The Plant Extract Yields

The percentage yields of the leaf extract of *M. oleifera* for both ethanolic and aqueous extracts were 32.2% and 24.8%, respectively. This revealed that ethanolic extract exhibited a higher yield percentage in comparison to aqueous extract. The pHs for both extracts were similar at 5.8 which is slightly acidic.

### 3.2. Preliminary Phytochemical Detection of the Ethanolic and Aqueous Extracts

Different active compounds were observed within the ethanolic and aqueous extracts as shown in Table 3. These included polyphenols as the major constituents, in addition to flavonoids, alkaloids, and tannins. Other components such as saponins and glycosides were also identified in both extracts but in lesser amounts.

### 3.3. Thin Layer Chromatography

TLC was performed of both the ethanolic and aqueous extracts for the estimation of phenolic compounds and flavonoids using different solvent systems (mobile phase). The identification of the active compounds was based on similarities in *R*_*f*_ values of separated compounds and standards.

**Figure 1:**
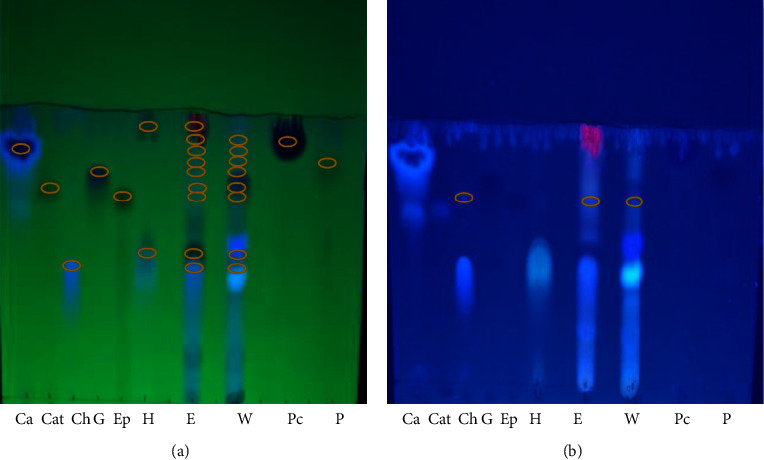
Thin layer chromatogram plate for phenolic compounds under UV 254 nm (a), 366 nm (b): caffeic acid (Ca), catechin (Cat), chlorogenic acid (Ch), gallic acid (G), epicatechin (Ep), hydroquinone (H), paracumaric acid (Pc), pyrogallol (P), ethanolic extract (E), and aqueous extract (W). Note: Spots indicating the presence of another isomer of chlorogenic acid can be visualized at wavelength of 366 nm.

**Figure 2:**
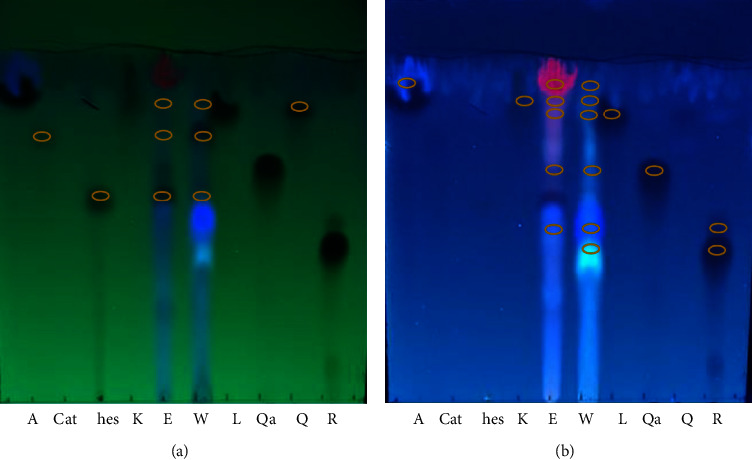
Thin layer chromatogram plates for flavonoids under UV 254 nm (a), 366 nm (b): apigenin (A), catechin (Cat), hesperitin (hes), kaempferol (K), luteolin (L), quarcitrin (Qa), quercetin (Q), rutin (R), ethanolic extract (E), and aqueous extract (W). Note: Spots indicating the presence of A, hes, and Q can be visualized at a wavelength of 254 nm.

The retardation factors of the phenolic compounds are shown in Table 4, and the corresponding TLC plate is shown in Figure 1. The retardation factors of the flavonoids appear in Table 5, with their TLC plate shown in Figure 2.

- All compounds were detected in accordance with their corresponding standards, and other unknown spots were also present in both ethanolic and aqueous extracts.

### 3.4. Qualitative and Quantitative Analyses Using HPLC

**Figure 3:**
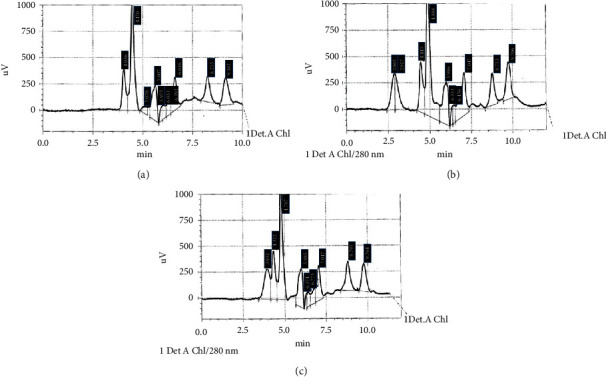
Chromatography profile of phenolic compounds. (a) Graph showing the peaks for standard phenolic acids: gallic acid (peak 4.036), vanillic acid (peak 4.470), catechin (peak 5.070), caffeic acid (peak 5.607), ferulic acid (peak 5.975 and 6.033), epicatechin (peak 6.392), chlorogenic acid (peak 6.616), P-coumaric acid (peak 8.255), and sinapic acid (peak 9.162). (b) Graph showing the phenolic compounds within the ethanolic extract: vanillic acid (peak 4.445), catechin (peak 4.888), ferulic acid (peak 5.986), epicatechin (peak 6.333), and chlorogenic acid (peak 6.456). (c) Graph showing the peaks of phenolic compounds in aqueous extracts: gallic acid (peak 3.932), vanillic acid (peak 4.322), catechin (peak 4.787), ferulic acid (peak 5.985), epicatechin (peak 6.374), and chlorogenic acid (peak 6.775).

After calculating the retention time, the area under the peak for the standard concentration (1.5 *μ*g/mL) of all standard phenolic compounds was used in this study as shown in the HPLC chromatogram (Figure 3(a)). These phenolic compounds were tested in both ethanolic and aqueous extracts, and their chromatograms are shown in Figures 3(b) and 3(c), respectively. The concentrations of phenolic compounds with similar peaks between the standards and extract solutions were calculated and are presented in Table 6.

The ethanolic and aqueous extracts were rich in many phenolic compounds including catechin, ferulic acid, epicatechin, caffeic acid, vanillic acid, and chlorogenic acid. While gallic acid was present in the aqueous extract, the ethanolic extract constituents did not contain gallic acid.

**Figure 4:**
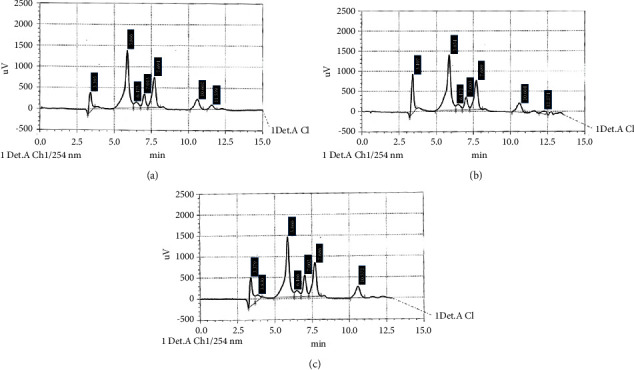
Chromatography profile of flavonoids. (a) Graph showing the peaks for standards of flavonoids: rutin (peak 3.395), caffeic acid (peak 5.866), apigenin (peak 6.475), hesperetin (peak 7.013), kaempferol (peak 7.694), coumarin (peak 10.602), and myricetin (peak 11.559). (b) Graph showing flavonoids within the ethanolic extract including rutin (peak 3.407), caffeic acid (peak 5.854), apigenin (peak 6.471), hesperetin (peak 7.003), kaempferol (peak 7.689), and coumarin (peak 10.604). (c) Graph showing the peaks of flavonoids in the aqueous extract: rutin (peak 3.379), caffeic acid (peak 5.860), apigenin (peak 6.466), hesperetin (peak 7.002), kaempferol (peak 7.686), and coumarin (peak 10.572).

After calculating the retention time, the area under the peak for standard concentrations (1 *μ*g/mL) of all flavonoids standards used in this study was as shown in the HPLC chromatogram in Figure 4(a). These flavonoids were tested in both ethanolic and aqueous extracts, and their chromatograms are shown in Figures 4(b) and 4(c), respectively. The concentrations of flavonoids with similar peaks between the standards and extract solutions were calculated and are presented in Table 7.

The ethanolic and aqueous extracts were rich in the following flavonoids: rutin, hesperetin, apigenin, kaempferol, and coumarin. However, myricetin was not detected in either of the extracts.

### 3.5. MIC and MBC Values for the Ethanolic and Aqueous Extracts against Selected Oral Pathogens

**Figure 5:**
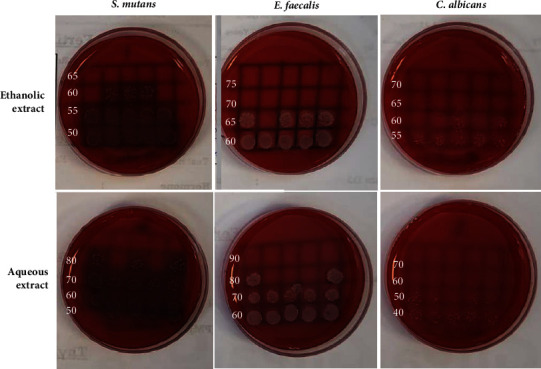
Blood agar plates for MBC value determination after 24 hours' incubation for the ethanolic and aqueous extracts against *Streptococcus mutans*, *Enterococcus faecalis*, and *Candida albicans*.

MIC and MBC mean values (*n* = 3) for both ethanolic and aqueous extracts against *Streptococcus mutans*, *Enterococcus faecalis*, and *Candida albicans* are presented in Table 8, while Figure 5 shows blood agar plates for MBC value determination after 24 hours of incubation for both ethanolic and aqueous extracts. Lower concentrations of the ethanolic extract were required for MIC and MBC of both *Streptococcus mutans* and *Enterococcus faecalis* in comparison to the aqueous extract. However, the aqueous extract required lower concentrations to obtain MIC and MBC values against *Candida albicans*.

### 3.6. Sensitivity Test

**Figure 6:**
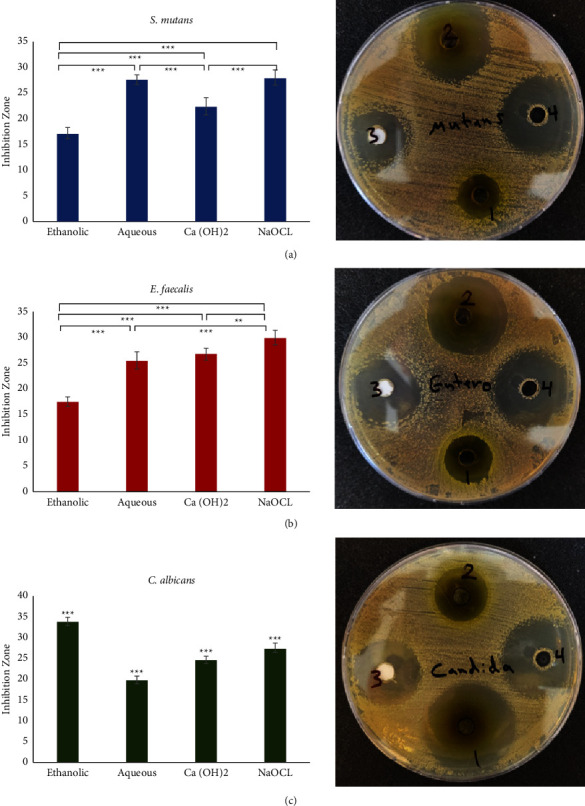
Sensitivity test of different microorganisms against ethanolic and aqueous extracts, NaOCl and Ca(OH)_2_. (a–c) The sensitivity tests of *S. mutans*, *E. faecalis*, and *C. albicans*, respectively. The *p* values presented were as follows: ^*∗*^*p* ≤ 0.05; ^*∗∗*^*p* ≤ 0.01; ^*∗∗∗*^*p* ≤ 0.001.

The mean values of the measured inhibition zones in (mm) of both the ethanolic and the aqueous extracts in comparison with calcium hydroxide and sodium hypochlorite against *Streptococcus mutans*, *Enterococcus faecalis*, and *Candida albicans* are illustrated in Figure 6. The aqueous extract showed a higher inhibition zone than the ethanolic extract for both *Streptococcus mutans* and *Enterococcus faecalis* (27.6 ±0 .9 and 25.6 ± 1.7, respectively). These values showed a statistically significant difference (*p* ≤ 0.001) for all tested materials except with NaOCl and Ca(OH)_2_ in *Streptococcus mutans* and *Enterococcus faecalis*, respectively. However, the ethanolic extract had a higher inhibition zone against *Candida albicans* with a statistically significant difference (*p* ≤ 0.001) for all tested materials.

## 4. Discussion

In this study, phytochemical screening and qualitative and quantitative analyses of *M. oleifera* leaf extracts were performed and their antimicrobial activities against certain root canal pathogens were identified.

The leaf extraction was done using two different techniques and solvents: cold maceration with ethanol and digestion extraction with water. Obtaining the percentage yield of extract is a particularly significant aspect in phytochemical extraction to assess the efficiency of the standard extraction for a particular plant, different parts of the same plant, or different solvents used [38]. The cold maceration method exhibited a higher yield of extract, which could be an effect of the extracting solvent nature, owing to the presence of various compounds with different chemical properties and polarities that may or may not be soluble in a particular solvent [39]. The differences between ethanolic and aqueous extract yields may be due to the efficiency variance of the extracting solvents in dissolving endogenous compounds from the plant material [40]. High temperature of processing conditions, on the other hand, may lead to losing parts of the natural antioxidants from extracts, as heat may accelerate oxidation and other degenerative reactions [39]. This could explain the decrease in the extract yield in aqueous extraction. Also, increasing drying temperature causes a degradation of phenolic compounds, with a significant reduction in the antioxidant activity of the extracts [41]. Hence, the preferred drying condition for the plant was in the shade, which is superior to drying in an oven or in the sun [42].

The results of the present study agree with the finding by Vongsak et al. [43] that maceration with 70% ethanol is the most efficient pharmaceutical method for *M. oleifera* leaves extraction, giving higher percentage yield, with the highest number of flavonoids and phenolic compounds and the most potent antioxidant activity. Chigurupati et al. [44] found that *Moringa* leaf extraction by maceration with 70% ethanol was convenient and cost-effective and produced more yield (about 14%). However, the result disagrees with the Muhammad et al.' [45] study which showed that the aqueous extract gave a higher percentage yield than that of the ethanolic extract. This difference could be due to the different geographical conditions in the places where the plant leaves were collected [46] and polarities of different compounds present in the leaves [47].

Phytochemicals are various groups of naturally occurring secondary metabolites that are biosynthesized by plants and have biological importance due to their vital role in the plant defense mechanism against different pathogenic microbes [48]. Studies that reported on the active phytochemicals of the *M. oleifera* plant were not uniform, and there was incompatibility in their reports. This is possibly due to the differences in season and agroclimatic locations of the plants [49], genetic impacts, cultivation, drying, and the method used for extraction [50]. It is preferable to identify the active ingredients when studying each medicinal plant due to the variation in the cultivation areas which could be associated with the presence of secondary metabolites of the plants in response to various environmental conditions. Such influence was reported in a study [51] which demonstrated that the height and biomass of the plant can be reduced in cases where water is lacking in comparison to ordinary conditions of cultivation, while glucosinolate quantity may be enhanced. Other factors, such as the type of solvent used and its concentration, the ratio of liquid to solid and particle size of the plant material, pH, temperature, and time, could have a significant influence on the efficacy of solvent extraction [52]. Polar solvents, for example, were utilized to extract polyphenols from plants [39].

The preliminary phytochemical analysis in this study confirmed the presence of alkaloids, flavonoids, glycosides, polyphenols, saponins, and tannins in both ethanolic and aqueous extracts of the leaves, with polyphenols and flavonoids being the most prominent detected compounds. The fact that these bioactive compounds were identified in leaves in great amounts may explain their pharmacological activity, as several *in vitro* and *in vivo* studies have confirmed antioxidants, anti-inflammatory, immunomodulatory, and anticancer properties of *M. oleifera* [53]. These results are in agreement with different previous studies that studied the phytochemical screening of ethanolic and aqueous *M. oleifera* extracts [26, 54–56]. While Patel et al. [57] indicated the presence of the same compounds in both ethanolic and aqueous extracts, but tannins were detected only in the ethanolic extract and glycoside was lacking in both extracts.

Phenolic acids are derived from hydroxybenzoic acid and hydroxycinnamic acid which are naturally present in plants, while flavonoids are synthesized by the plant in reaction to microbial infections, with a benzo-*γ*-pyrone ring as a common structure [58]. Phenolic compounds represent the largest group of plant secondary metabolites and are valued for their anti-inflammatory, antihepatotoxic, and antioxidant properties and free radical scavengers [59]. These compounds have an inhibitory effect on microorganism growth which is proportional to the content of phenolic compounds in the plant extract [60]. Therefore, the TLC and HPLC analyses focused on the detection of these compounds in both extracts.

Thin layer chromatography (TLC) is an easy, inexpensive, rapid, and commonly utilized method to analyze and isolate small organic natural and synthetic products [61]. In the current study, TLC analysis gave a significant result indicating the presence of a number of important phenolic and flavonoid compounds. The most suitable solvent system was found to be chloroform : glacial acetic acid : methanol (4 : 5 : 1), which provided the best separation of the active compounds. This might be due to its polarity which was able to provide solubility and balancing of the sample affinity for the solvent and the stationary phase to accomplish the separation of compounds within samples [62]. The results of the present study are in agreement with Chauhan et al. [21] who also used TLC to demonstrate the presence of caffeic acid, chlorogenic acid, gallic acid, and quercetin and obtained similar findings to this study. In addition, Marrufo et al. [63] found that both aqueous and ethanolic extracts demonstrated the presence of catechin, epicatechin, kaempferol, and quercetin.

HPLC can be applied for the separation, identification, and quantification of the compounds present in extracts such as polyphenols [64]. Abd Rani et al. [65] identified different flavonoids and phenolic compounds which can be detected within *M. oleifera* leaf extracts. Hence, markers of these compounds were used in the present study. The HPLC results revealed that both ethanolic and aqueous extracts contained various types of phenolic and flavonoid compounds. HPLC analysis was performed under specific conditions and at two specific wavelengths: 280 nm for the phenolic compounds and 254 nm for the flavonoids, specific mobile and stationary phases. Therefore, not all chemical compounds in the ethanolic and aqueous extracts were detected. The measured compounds were only those that separated under the HPLC conditions provided and had optimal absorbance at these wavelengths. The different environmental conditions in different countries, i.e., in temperature, rainfall, sunlight, soil characteristics, and altitude [66], in addition to differences in the harvesting season, plant genetics, maturity of the leaf, and the drying and extraction method may be the reasons for some differences in the range of values reported in this study in comparison with other similar studies [53]. Furthermore, variations in the polarity of solvents and their diffusion strengths, the structural complexity, and selective solubility of secondary metabolites in a particular solvent may explain such variation in phenolic and flavonoid contents within an extraction solvent [67].

The results are in agreement with Karthivashan et al. [68] as they demonstrated the presence of flavonoids: apigenin, kaempferol, and quercetin, in the 90% ethanolic extract of *M. oleifera* leaves. Another study [69] revealed the presence of chlorogenic acid, ferulic acid, gallic acid, and p-coumaric acid in both aqueous and ethanolic extracts. Also, Muzammil et al. [41] demonstrated a similar finding but with the addition of p-coumaric acid and sinapic acid which were not detected in the current study. These differences may be due to factors such as the development stage of leaves and handling at the time of harvesting, genetic variance, and different agroclimatic conditions [70].

However, the results showed that the aqueous extract contained a larger quantity of flavonoids than those within the ethanolic extract. This is in agreement with different studies [71–73] which demonstrated that water acts as a strong extraction medium capable of dissolving most of the phenolic and flavonoid compounds. While Nobossé et al. [67] concluded that ethanol was a more efficient solvent for extracting high flavonoid content exhibiting higher antioxidant activity for *M. oleifera* leaf compared to aqueous extract.

For antimicrobial effect assessments, resazurin microtiter assay was selected since it is a simple, sensitive, and reliable method, which can give fast results and at considerably low cost [74, 75].

Both ethanolic and aqueous extracts proved to possess antimicrobial activities against *S. mutans*, *E. faecalis*, and *C. albicans in vitro* well diffusion assay. These antimicrobial actions could be attributed to flavonoids, saponins, tannins, and a number of several phenolic compounds. In addition, there is the presence of substantial amounts of protein and fatty acids [76]. Fatty acids with short and long carbon chains exhibited antimicrobial activities against Gram-negative and Gram-positive bacteria [77]. At the molecular level, flavonoids can complex with proteins via nonspecific forces such as hydrogen bonds, hydrophobic effects, and covalent bond formation. Consequently, these bioactive compounds could inactivate the adhesions of the microbes, enzymes, and cell envelope transport proteins and may be able to disrupt microbial membranes [78]. Also, phenolic compounds hold an active hydroxyl group that permits the phenols to engage in hydrogen bonding with bacterial membranes which result in membrane disruption that can lead to inhibition of membrane transport, failure to sustain pH gradient, and improper regulation of the ATP level [79]. Moreover, it was reported that antibacterial ability will increase as pH decreases [80]. The pH values of these extracts were slightly acidic, hence another cause for antimicrobial action.

*S. mutans* are Gram-positive facultative anaerobic bacteria, which play an important role in the development of oral biofilm through the production of extracellular polysaccharides [77]. These bacteria have the ability to synthesize considerable quantities of glucan from dietary sucrose, providing binding sites for cariogenic bacterial colonization on the tooth surface [81]. Moreover, *S. mutans* strains were found in inflamed pulp at a high prevalence in both asymptomatic and symptomatic endodontic infections [82, 83]. According to the results of this study, *M. oleifera* leaf extracts showed antibacterial activity against *S. mutans.* This is in agreement with a previous study done by Elgamily et al. [84]. The activity of flavonoids in the inhibition of *S. mutans*, such as catechins, may be due to complexing activities [85] and apigenin through increasing *S. mutans* membrane proton permeability and inhibiting bacterial acid production [86].

*E. faecalis* bacteria are responsible for multiple oral illnesses, such as dental abscess, apical periodontitis, and persistent endodontic infections [87], and considered the most prevalent bacteria in cases of endodontic treatment failure [10, 88]. These bacteria can form biofilms in aerobic, anaerobic, abundant, or insufficient nutrition environments [88] and can resist chemomechanical root canal preparation and are antiseptic [87]. This study showed that the antimicrobial effects of ethanolic and aqueous extracts of *M. oleifera* against *E. faecalis* were similar to those of 3% sodium hypochlorite. This antibacterial activity is in agreement with previous studies [12, 54]. This is possibly due to the presence of flavonoids in the extracts, namely, apigenin, rutin, and luteolin which have antibacterial activity against *E. faecalis* [86].

*C. albicans*, a fungus that can colonize the root canal's dentinal wall, penetrate the dentinal tubules, and form biofilms [89], as reported in persistent posttreatment apical periodontitis, can switch between blastospore and hyphal form and thus can invade the host tissue and avoid phagocytosis by macrophages and resist a wide range of pH and harsh environments allowing it to cause persistent infection [90]. The results of the current study also demonstrated an antifungal effect of *M. oleifera* leaf extracts against *C. albicans* which is in agreement with previous reports [91–93]. The antifungal activity could be explained by the ability of phenolic compounds to disrupt the homeostasis of Ca^+2^ and H^+^ ions, upregulation and downregulation of gene transcription, breakdown of membrane integration, and impairment of the biosynthesis of ergosterol in *C. albicans* [94]. Flavonoids such as luteolin, quercetin, and rutin also possess inhibition activity against *C. albicans* [86]. However, the current results disagree with Moyo et al. [95] and Patel et al. [57] who found no antifungal activity of *M. oleifera* aqueous and ethanolic extracts against *C. albicans*. This difference is possibly due to the variation in the chemical composition and quantity of compounds in the extracts of the current study as a result of differences in environments from which the plant leaves were gathered, the season, and the physiological status of the plant [95].

Differences in the range of MICs between the results of this study and other studies may be attributed to many factors such as temperature, inoculation size, and microorganism type used [96]. In addition, the properties of extracts are influenced by several factors, such as plant components and fresh or dried varieties, which are in turn affected by climate, harvesting time, extraction method, solvent type, and stability of components [97]. Another plausible reason could be the use of Iraqi-isolated bacterial species that may be more resistant to antimicrobial agents [98]. In particular, the high prevalence of antimicrobial resistance in Iraq may be related to the unnecessary use of antibiotics [99].

Although this study used two common extraction methods which were easy and simple, there are other extraction protocols with a range of technologies which could have revealed the presence of different content within *M. oleifera* leaf [100]. Also, this study investigated the antimicrobial function of *M. oleifera* leaf extracts through the *in vitro* well diffusion method since there are differences in the microbial resistance between planktonic and biofilms against antimicrobial agents [77]. Further studies are required to investigate the antibiofilm action and cytotoxicity of these extracts and each of their active components and their possible adverse reactions.

## 5. Conclusion

Ethanolic and aqueous extracts of *M. oleifera* leaves that were cultivated in Baghdad and evaluated in this study contained considerable quantities of medicinal phytochemicals, particularly flavonoids and phenolic compounds as confirmed by TLC and HPLC tests. These extracts demonstrated powerful antimicrobial activities against different endodontic pathogens, including *S. mutans*, *E. faecalis*, and *C. albicans*, and this property can be employed in the construction of useful endodontic treatment materials in gel or solution form, as irrigation solutions or intracanal medication. Although these *in vitro* results were encouraging, further isolation and characterization of individual active constituents from these extracts and identification of their antimicrobial properties, evaluation of the extracts' anti-inflammatory effect, biocompatibility, and safety must be performed in preclinical and clinical research studies to confirm the possible utilization of this plant and its derivatives for endodontic treatments.

## Figures and Tables

**Table 1:** HPLC conditions for phenolic compounds.

Instrument	Shimadzu, Japan

Mobile phase	(A) Solution: 0.1% formic acid (B) Solution acetonitrile : methanol : water 80% : 10% : 10% in the ratio of 6% A : B
Particle size	5 *μ*m
Column	ODS C_18_ (250 mm × 4.6 mm internal diameter)
Flow rate	1.2 mL/min
Column temperature	Room temperature
Injection volume	20 *μ*L
Injection concentration	1.5 *μ*g/mL
Detection wavelength	UV-Vis at wavelength of 280 nm
Standards used	Gallic acid, vanillic acid, catechin, caffeic acid, ferulic acid, epicatechin, chlorogenic acid, P-coumaric acid, and sinapic acid

**Table 2:** HPLC conditions for flavonoids.

Instrument	Shimadzu, Japan

Mobile phase	(A) solution: 1% acetic acid (B) Solution : acetonitrile : water 80% in ratio of 90% B : A
Particle size	5 *μ*m
Column	ODS C_18_ (250 mm × 4.6 mm internal diameter)
Flow rate	1.2 mL/min
Column temperature	Room temperature
Injection volume	20 *μ*L
Injection concentration	1 *μ*g/mL
Detection wavelength	UV-Vis at wavelength of 254 nm
Standards used	Rutin, caffeic acid, apigenin, hesperetin, kaempferol, coumarin, and myricetin

**Table 3:** Active compounds detected within ethanolic and aqueous extracts of *M. oleifera*.

Active compounds	Ethanolic	Aqueous
Alkaloids	++	++
Flavonoids	++	++
Glycosides	+	+
Polyphenols	+++	+++
Saponins	+	+
Tannins	++	++

(+) indicates the presence of the component in the plant. ++ indicates moderate presence. +++ indicates intense presence.

**Table 4:** Retardation factor results for the phenolic compounds.

Compound	RF values	Ethanolic extract	Aqueous extract
Catechin Cat	0.7241	0.7241	0.7241
Caffeic acid Ca	0.8620	0.8620	0.8620
Chlorogenic acid Ch1	0.4827	0.4827	0.4827
Chlorogenic acid Ch2	0.7356	0.7356	0.7356
Gallic acid G	0.8045	0.8045	0.8045
Epicatechin Ep	0.7412	0.7412	0.7412
Hydroquinone H1	0.5747	0.5747	0.5747
Hydroquinone H2	0.97701	0.97701	—
Paracumaric acid Pc	0.9310	0.9310	0.9310
Pyrogallol P	0.8505	0.8505	0.8505
Other phenolic compounds in the plant extracts		0.29	0.27

**Table 5:** Retardation factor results for the flavonoids.

Compound	RF values	Ethanolic extract	Aqueous extract
Apigenin A	0.8785	0.8785	0.8785
Catechin Cat	0.7570	0.7570	0.7570
Hesperitin hes	0.5887	0.5887	0.5887
Kaempferol K	0.8598	0.8598	0.8598
Luteolin L	0.8224	0.8224	0.8224
Quarcitrin Qa	0.7196	0.7196	0.7196
Quercetin Q	0.8504	0.8504	0.8504
Rutin R1	0.4672	—	0.4672
Rutin R2	0.5327	0.5327	0.5327
Other flavonoids in the plant extracts		0.3, 0.43, 0.9	0.3, 0.43

**Table 6:** HPLC results showing retention time (min), area under the curve, and the concentrations (*μ*g/mL) of phenolic compounds for ethanolic and aqueous extracts.

Name of phenolic compound	Aqueous extract			Ethanolic extract		
	Retention time (in minutes)	Area	Concentration (*μ*g/mL)	Retention time (in minutes)	Area	Concentration (*μ*g/mL)
Gallic acid	3.932	7004	2.1635	Not found	—	—
Vanillic acid	4.322	6920	0.7463	4.445	7305	0.7879
Catechin	4.787	15765	20.1598	4.888	23604	30.1841
Caffeic acid	Not found	—	—	Not found	—	—
Ferulic acid	5.985	7121	8.8058	5.986	9697	11.9913
Epicatechin	6.374	1687	1.402	6.333	1351	1.1233
Chlorogenic acid	6.775	2321	0.7351	6.456	1477	0.4677
P-Coumaric acid	Not found	—	—	Not found	—	—
Sinapic acid	Not found	—	—	Not found	—	—

**Table 7:** HPLC results showing retention time (min), area under the curve, and the concentrations (*μ*g/mL) of flavonoids for ethanolic and aqueous extracts.

Name of flavonoid compound	Aqueous extract			Ethanolic extract		
	Retention time (in minutes)	Area	Concentration (*μ*g/mL)	Retention time (in minutes)	Area	Concentration (*μ*g/mL)
Rutin	3.379	9198	1.6501	3.407	10326	1.8525
Caffeic acid	5.860	29568	1.076	5.854	29870	1.087
Apigenin	6.466	3336	1.1323	6.471	2861	0.9711
Hesperetin	7.002	7793	1.6144	7.003	4989	1.0335
Kaempferol	7.686	14335	1.1049	7.689	12899	0.9942
Coumarin	10.572	6081	1.1068	10.604	5141	0.9357
Myricetin	Not found	—	—	Not found	—	—

**Table 8:** Values of MIC and MBC.

Name of microorganism	Ethanolic extract		Aqueous extract	
	MIC (mg/mL)	MBC (mg/mL)	MIC (mg/mL)	MBC (mg/mL)
*Streptococcus mutans*	60	65	70	80
*Enterococcus faecalis*	65	70	80	90
*Candida albicans*	55	65	50	60

## Data Availability

The data supporting the findings of this study are included within the article.

## References

[1] Karobari M. I., Adil A. H., Assiry A. A. (2022). Herbal medications in endodontics and its application—a review of literature. *Materials*.

[2] Abdull Razis A. F., Ibrahim M. D., Kntayya S. B. (2014). Health benefits of Moringa oleifera. *Asian Pacific Journal of Cancer Prevention*.

[3] Saini R. K., Sivanesan I., Keum Y.-S. (2016). Phytochemicals of Moringa oleifera: a review of their nutritional, therapeutic and industrial significance. *3 Biotech*.

[4] Alegbeleye O. O. (2018). How functional is Moringa oleifera? A review of its nutritive, medicinal, and socioeconomic potential. *Food and Nutrition Bulletin*.

[5] Diod P. S. (2020). Standarization based on chemical markers of Moringa oleifera herbal products using bioautography assay, thin layer chromatography and high performance liquid chromatography-diode array detector. *Malaysian Journal of Analytical Sciences*.

[6] Alghamdi F., Shakir M. (2020). The influence of *Enterococcus faecalis* as a dental root canal pathogen on endodontic treatment: a systematic review. *Cureus*.

[7] Almadi E. M., Almohaimede A. A. (2018). Natural products in endodontics. *Saudi Medical Journal*.

[8] Agrawal V., Kapoor S., Agrawal I. (2017). Critical review on eliminating endodontic dental infections using herbal products. *Journal of Dietary Supplements*.

[9] Sinha D. J., Sinha A. A. (2014). Natural medicaments in dentistry. *Ayu*.

[10] Al-Badr R. J., Al-Huwaizi H. F. (2017). Effect of tea tree, thymus vulgaris and nigella sativa oils on the elimination of Enterococcus faecalis (in vitro study). *Journal of Baghdad College of Dentistry*.

[11] Shafiq N. E., Mahdee A. F. (2023). Moringa oleifera use in maintaining oral health and its potential use in regenerative dentistry. *The Scientific World Journal*.

[12] Arévalo-Híjar L., Aguilar-Luis M. A., Caballero-García S., Gonzáles-Soto N., Del Valle-Mendoza J. (2018). Antibacterial and cytotoxic effects of Moringa oleifera (Moringa) and Azadirachta indica (Neem) methanolic extracts against strains of *Enterococcus faecalis*. *International journal of dentistry*.

[13] Ashraf K., Noushad M., Suneetha M. (2020). Antibacterial efficacy of muringa seed extract and potato peel extract against *Enterococcus faecalis*. *Contemporary Clinical Dentistry*.

[14] Sopandani P., Iskandar B., Ariwibowo T., Djamil M. (2020). Antibacterial effects of moringa oleifera leaf extract against enterococcus faecalis in vitrio. *Scientific Dental Journal*.

[15] Onduru Okeyo G., Charimbu M. K., Nyaanga J., Mendes T. (2022). Antibacterial activity of guava, moringa, camphor bush and pelargonium extracts against bacterial wilt (Ralstonia pseudosolanacearum sp. nov.) of potato. *Saudi Journal of Biological Sciences*.

[16] Ibrahim N., Kebede A. (2020). In vitro antibacterial activities of methanol and aqueous leave extracts of selected medicinal plants against human pathogenic bacteria. *Saudi Journal of Biological Sciences*.

[17] Abubakar A. R., Haque M. (2020). Preparation of medicinal plants: basic extraction and fractionation procedures for experimental purposes. *Journal of Pharmacy and BioAllied Sciences*.

[18] Kaushik S., Dar L., Kaushik S., Yadav J. P. (2021). Anti-dengue activity of super critical extract and isolated oleanolic acid of Leucas cephalotes using in vitro and in silico approach. *BMC Complementary Medicine and Therapies*.

[19] Shri Chengama Raju N., Wing Kei W. (2022). Anti-angiogenic screening of moringa oleifera leaves extract using chorioallantonic membrane assay. *Iraqi Journal of Pharmaceutical Sciences*.

[20] Al-Halbosiy M. M. F., Hasan Z. Y. M., Mohammad F. I., Abdulhameed B. A. (2020). Biological activities of Iraqi fig (ficuscarica) CrudeEthanolic and total flavonoids extracts. *Iraqi Journal of Science*.

[21] Chauhan A. P., Patel D. M., Pate J. D., Singh N. K. (2021). Extraction, screening, and characterization of bioactive compounds from moringa oleifera: extends life-span of *Caenorhabditis elegans*. *Current Trends in Biotechnology and Pharmacy*.

[22] Pandey A., Tripathi S. (2014). Concept of standardization, extraction and pre phytochemical screening strategies for herbal drug. *Journal of Pharmacognosy and Phytochemistry*.

[23] Muttalib L. Y., Naqishbandi A. M. (2012). Antibacterial and phytochemical study of Iraqi Salvia officinalis leave extracts. *Iraqi Journal of Pharmaceutical Sciences*.

[24] Khudhair N. (2016). Biochemical and histopathollogical study of moringa oleifera extract on the fertility in male mic.

[25] Jee C.-H. (2020). Preliminary phytochemical screening and thin layer chromatography of selected extract of Moringa oleifera leaf. *International journal of chemical studies*.

[26] Ameerah Shaeroun A. R., Hamed Alqamoudy A. B. A., Mohamed K. S., Nouri Kushlaf N. A., Akram Almabrouk misbah A. M. E.-M., Zuhur rajab Almes S. T. O. (2019). Thin layer chromatography (TLC) and phytochemical analysis of moringa oleifera methanol, ethanol, water and Ethyl acetate extracts. *Saudi Journal of Medical and Pharmaceutical Sciences*.

[27] Sharma V., Paliwal R. (2013). Preliminary phytochemical investigation and thin layer chromatography profiling of sequential extracts of Moringa oleifera pods. *International Journal of Green Pharmacy*.

[28] Kupiec T. (2004). Quality-control analytical methods: high-performance liquid chromatography. *International Journal of Pharmaceutical Compounding*.

[29] Mahdi A., Al-Huwaizi H., Abbas I. (2018). A comparative evaluation of antimicrobial activity of the ethanolic extract of Cinnamomum zeylanicum and NaOCl against oral pathogens and against swabs taken from nonvital teeth-An in vitro study. *International Journal of PharmTech Research*.

[30] Balouiri M., Sadiki M., Ibnsouda S. K. (2016). Methods for in vitro evaluating antimicrobial activity: a review. *Journal of Pharmaceutical Analysis*.

[31] Martin A., Camacho M., Portaels F., Palomino J. C. (2003). Resazurin microtiter assay plate testing of *Mycobacterium tuberculosis* susceptibilities to second-line drugs: rapid, simple, and inexpensive method. *Antimicrobial Agents and Chemotherapy*.

[32] Elshikh M., Ahmed S., Funston S. (2016). Resazurin-based 96-well plate microdilution method for the determination of minimum inhibitory concentration of biosurfactants. *Biotechnology Letters*.

[33] Singh C. R., Kathiresan K., Anandhan S., Suganthi K. (2014). Antioxidant and antibacterial activity of field grown and tissue cultured root callus of mangrove species. *European Journal of Medicinal Plants*.

[34] Loo Y. Y., Rukayadi Y., Nor-Khaizura M. A. (2018). In vitro antimicrobial activity of green synthesized silver nanoparticles against selected gram-negative foodborne pathogens. *Frontiers in Microbiology*.

[35] Jang J.-H., Kim Y.-I., Lee H. (2014). Antimicrobial activity of Prunus mume extract to oral microbes. *Journal of Korean Society of Dental Hygiene*.

[36] Prastiyanto M. E., Tama P. D., Ananda N., Wilson W., Mukaromah A. H. (2020). Antibacterial potential of jatropha sp. latex against multidrug-resistant bacteria. *International Journal of Microbiology*.

[37] Jayasuriya W., Jayaweera N., Adurapotha H., Meedin F., Uluwaduge D., Arawwawala L. (2021). Antimicrobial effect of polyphenols enriched fractions of moringa oleifera lam. Leaves at the flowering stage against microbial strains causing oral and wound infections. *Pharmaceutical Journal of Sri Lanka*.

[38] Garg P., Garg R. (2019). Phytochemical screening and quantitative estimation of total flavonoids of Ocimum sanctum in different solvent extract. *The Pharma Innovation Journal*.

[39] Sultana B., Anwar F., Ashraf M. (2009). Effect of extraction solvent/technique on the antioxidant activity of selected medicinal plant extracts. *Molecules*.

[40] Siddhuraju P., Becker K. (2003). Antioxidant properties of various solvent extracts of total phenolic constituents from three different agroclimatic origins of drumstick tree (Moringa oleifera Lam.) leaves. *Journal of Agricultural and Food Chemistry*.

[41] Muzammil S., Neves Cruz J., Mumtaz R. (2023). Effects of drying temperature and solvents on in vitro diabetic wound healing potential of moringa oleifera leaf extracts. *Molecules*.

[42] Thamer F., Dauqan E., Naji K. (2018). The effect of drying temperature on the antioxidant activity of thyme extracts. *Journal of Food Technology and Preservation*.

[43] Vongsak B., Sithisarn P., Mangmool S., Thongpraditchote S., Wongkrajang Y., Gritsanapan W. (2013). Maximizing total phenolics, total flavonoids contents and antioxidant activity of Moringa oleifera leaf extract by the appropriate extraction method. *Industrial Crops and Products*.

[44] Chigurupati S., Al-Murikhy A., Almahmoud S. A. (2022). Molecular docking of phenolic compounds and screening of antioxidant and antidiabetic potential of Moringa oleifera ethanolic leaves extract from Qassim region, Saudi Arabia. *Saudi Journal of Biological Sciences*.

[45] Muhammad A. A., Pauzi N. A. S., Arulselvan P., Abas F., Fakurazi S. (2013). In vitro wound healing potential and identification of bioactive compounds from Moringa oleifera Lam. *BioMed Research International*.

[46] Anokwuru C., Anyasor G., Ajibaye O., Fakoya O., Okebugwu P. (2011). Effect of extraction solvents on phenolic, flavonoid and antioxidant activities of three nigerian medicinal plants. *Nature and Science*.

[47] Barchan A., Bakkali M., Arakrak A., Pagán R., Laglaoui A. (2014). The effects of solvents polarity on the phenolic contents and antioxidant activity of three Mentha species extracts. *International Journal of Current Microbiology and Applied Sciences*.

[48] Verma A. K., Singh S. (2020). Phytochemical analysis and in vitro cytostatic potential of ethnopharmacological important medicinal plants. *Toxicology Reports*.

[49] Iqbal S., Bhanger M. (2006). Effect of season and production location on antioxidant activity of Moringa oleifera leaves grown in Pakistan. *Journal of Food Composition and Analysis*.

[50] Aliyu A., Shaari M. R., Ahmad Sayuti N. S. (2021). Moringa oleifera hydorethanolic leaf extract induced acute and sub-acute hepato-nephrotoxicity in female ICR-mice. *Science Progress*.

[51] Förster N., Ulrichs C., Schreiner M., Arndt N., Schmidt R., Mewis I. (2015). Ecotype variability in growth and secondary metabolite profile in Moringa oleifera: impact of sulfur and water availability. *Journal of Agricultural and Food Chemistry*.

[52] Cacace J., Mazza G. (2003). Optimization of extraction of anthocyanins from black currants with aqueous ethanol. *Journal of Food Science*.

[53] Leone A., Spada A., Battezzati A., Schiraldi A., Aristil J., Bertoli S. (2015). Cultivation, genetic, ethnopharmacology, phytochemistry and pharmacology of Moringa oleifera leaves: an overview. *International Journal of Molecular Sciences*.

[54] Das P. E., Abu-Yousef I. A., Majdalawieh A. F., Narasimhan S., Poltronieri P. (2020). Green synthesis of encapsulated copper nanoparticles using a hydroalcoholic extract of Moringa oleifera leaves and assessment of their antioxidant and antimicrobial activities. *Molecules*.

[55] Elangovan M., Rajalakshmi A., Jayachitra A., Mathi P., Bhogireddy N. (2014). Analysis of phytochemicals, antibacterial and antioxidant activities of Moringa oleifera Lam. leaf extract-An in vitro study. *International Journal of Drug Development & Research*.

[56] Nazmy S., Hassan B., Nihad A., Abeer E., Abd Elhamid E. M., Farid M. (2016). Biochemical studies on Moringa oleifera leaves extract. *Journal of Biology, Agriculture and Healthcare*.

[57] Patel P., Patel N., Patel D., Desai S., Meshram D. (2014). Phytochemical analysis and antifungal activity of Moringa oleifera. *International Journal of Pharmacy and Pharmaceutical Sciences*.

[58] Vergara-Jimenez M., Almatrafi M. M., Fernandez M. L. (2017). Bioactive components in Moringa oleifera leaves protect against chronic disease. *Antioxidants*.

[59] A Hussein R., A El-Anssary A. (2019). Plants secondary metabolites: the key drivers of the pharmacological actions of medicinal plants. *Herbal medicine*.

[60] Yang S.-Y., Kang M.-K. (2020). Biocompatibility and antimicrobial activity of Reynoutria elliptica extract for dental application. *Plants*.

[61] Gibbons S. (2012). An introduction to planar chromatography and its application to natural products isolation. *Natural Products Isolation*.

[62] Cai L. (2014). Thin layer chromatography. *Current Protocols Essential Laboratory Techniques*.

[63] Marrufo T., Encarnação S., Silva O. M. D. (2013). Chemical characterization and determination of antioxidant and antimicrobial activities of the leaves of Moringa oleifera. *International Network Environmental Management Conflicts*.

[64] Fanta Yadang S. A., Taiwe Sotoing G., Ngatcha Zouakeu K. S. (2019). Quantification of bioactive compounds and evaluation of the antioxidant activity of carissa edulis valh (apocynaceae) leaves. *The Scientific World Journal*.

[65] Abd Rani N. Z., Husain K., Kumolosasi E. (2018). Moringa genus: a review of phytochemistry and pharmacology. *Frontiers in Pharmacology*.

[66] Chikezie P. C., Ibegbulem C. O., Mbagwu F. N. (2015). Bioactive principles from medicinal plants. *Research Journal of Phytochemistry*.

[67] Nobossé P., Fombang E. N., Mbofung C. M. (2018). Effects of age and extraction solvent on phytochemical content and antioxidant activity of fresh Moringa oleifera L. leaves. *Food Science and Nutrition*.

[68] Karthivashan G., Tangestani Fard M., Arulselvan P., Abas F., Fakurazi S. (2013). Identification of bioactive candidate compounds responsible for oxidative challenge from hydro‐ethanolic extract of Moringa oleifera leaves. *Journal of Food Science*.

[69] Khalid S., Arshad M., Mahmood S. (2023). Extraction and quantification of moringa oleifera leaf powder extracts by HPLC and FTIR. *Food Analytical Methods*.

[70] Pakade V., Cukrowska E., Chimuka L. (2013). Metal and flavonol contents of Moringa oleifera grown in South Africa. *South African Journal of Science*.

[71] Owusu-Ansah M., Achel D. G., Adaboro R. M., Asare D. K., Amoatey H. M. (2011). Total phenolic content and antioxidant activity in leaf samples of twelve accessions of Moringa oleifera Lam. *International Journal of Chemical and Analytical Science*.

[72] El Sohaimy S. A., Hamad G. M., Mohamed S. E., Amar M. H., Al-Hindi R. R. (2015). Biochemical and functional properties of Moringa oleifera leaves and their potential as a functional food. *Global Advanced Research Journal of Agricultural Science*.

[73] García-Beltrán J. M., Mansour A. T., Alsaqufi A. S., Ali H. M., Esteban M. Á. (2020). Effects of aqueous and ethanolic leaf extracts from drumstick tree (Moringa oleifera) on gilthead seabream (*Sparus aurata* L.) leucocytes, and their cytotoxic, antitumor, bactericidal and antioxidant activities. *Fish & Shellfish Immunology*.

[74] Sarker S. D., Nahar L., Kumarasamy Y. (2007). Microtitre plate-based antibacterial assay incorporating resazurin as an indicator of cell growth, and its application in the in vitro antibacterial screening of phytochemicals. *Methods*.

[75] Monteiro M. C., de la Cruz M., Cantizani J. (2012). A new approach to drug discovery: high-throughput screening of microbial natural extracts against Aspergillus fumigatus using resazurin. *SLAS Discovery*.

[76] Teixeira E. M. B., Carvalho M. R. B., Neves V. A., Silva M. A., Arantes-Pereira L. (2014). Chemical characteristics and fractionation of proteins from Moringa oleifera Lam. leaves. *Food Chemistry*.

[77] Jwa S.-K. (2019). Efficacy of moringa oleifera leaf extracts against cariogenic biofilm. *Preventive Nutrition and Food Science*.

[78] Kumar S., Pandey A. K. (2013). Chemistry and biological activities of flavonoids: an overview. *The Scientific World Journal*.

[79] Banks J. M., Brandini D. A., Barbosa D. B. (2022). Leveraging microbicidal and immunosuppressive potential of herbal medicine in oral diseases. *Herbal Medicines*.

[80] Saghiri M. A., Delvarani A., Mehrvarzfar P. (2011). The impact of pH on cytotoxic effects of three root canal irrigants. *The Saudi Dental Journal*.

[81] Lee S.-H., Choi B.-K., Kim Y.-J. (2012). The cariogenic characters of xylitol-resistant and xylitol-sensitive Streptococcus mutans in biofilm formation with salivary bacteria. *Archives of Oral Biology*.

[82] Nomura R., Ogaya Y., Nakano K. (2016). Contribution of the collagen-binding proteins of Streptococcus mutans to bacterial colonization of inflamed dental pulp. *PLoS One*.

[83] Lima A. R., Herrera D. R., Francisco P. A. (2021). Detection of Streptococcus mutans in symptomatic and asymptomatic infected root canals. *Clinical Oral Investigations*.

[84] Elgamily H., Moussa A., Elboraey A., El-Sayed H., Al-Moghazy M., Abdalla A. (2016). Microbiological assessment of Moringa oleifera extracts and its incorporation in novel dental remedies against some oral pathogens. *Open access Macedonian journal of medical sciences*.

[85] Cowan M. M. (1999). Plant products as antimicrobial agents. *Clinical Microbiology Reviews*.

[86] Gutiérrez-Venegas G., Gómez-Mora J. A., Meraz-Rodríguez M. A., Flores-Sánchez M. A., Ortiz-Miranda L. F. (2019). Effect of flavonoids on antimicrobial activity of microorganisms present in dental plaque. *Heliyon*.

[87] Benbelaïd F., Khadir A., Abdoune M. A., Bendahou M., Muselli A., Costa J. (2014). Antimicrobial activity of some essential oils against oral multidrug–resistant *Enterococcus faecalis* in both planktonic and biofilm state. *Asian Pacific Journal of Tropical Biomedicine*.

[88] Zand V., Lotfi M., Soroush M. H., Abdollahi A. A., Sadeghi M., Mojadadi A. (2016). Antibacterial efficacy of different concentrations of sodium hypochlorite gel and solution on *Enterococcus faecalis* biofilm. *Iranian Endodontic Journal*.

[89] Yoo Y. J., Kim A. R., Perinpanayagam H., Han S. H., Kum K. Y. (2020). Candida albicans virulence factors and pathogenicity for endodontic infections. *Microorganisms*.

[90] Thienngern P., Panichuttra A., Ratisoontorn C., Aumnate C., Matangkasombut O. (2022). Efficacy of chitosan paste as intracanal medication against *Enterococcus faecalis* and Candida albicans biofilm compared with calcium hydroxide in an in vitro root canal infection model. *BMC Oral Health*.

[91] Mahdi L. H., Shafiq S. A., Galoob A. K., Muslem S. N. (2014). Effect of plant extracted Moringa Oeifera Lam on some isolated pathogens from mouth and teeth. *World Journal of Pharmaceutical Research*.

[92] Bagheri G., Martorell M., Ramí­rez-Alarcón K., Salehi B., Sharifi-Rad J. (2020). Phytochemical screening of Moringa oleifera leaf extracts and their antimicrobial activities. *Cellular and Molecular Biology*.

[93] Das P. E., Majdalawieh A. F., Abu-Yousef I. A., Narasimhan S., Poltronieri P. (2020). Use of A Hydroalcoholic extract of moringa oleifera leaves for the green synthesis of bismuth nanoparticles and evaluation of their anti-microbial and antioxidant activities. *Materials*.

[94] El Sohaimy S., Masry S. (2014). Phenolic Content, Antioxidant and Antimicrobial Activities of Egyptian and Chinese Propolis. https://www.researchgate.net/publication/269635744_Phenolic_Content_Antioxidant_and_Antimicrobial_Activities_of_Egyptian_and_Chinese_Propolis.

[95] Moyo B., Masika P. J., Muchenje V. (2012). Antimicrobial activities of Moringa oleifera Lam leaf extracts. *African Journal of Biotechnology*.

[96] Rahnama Vosough P., Habibi Najafi M. B., Edalatian Dovom M. R., Javadmanesh A., Mayo B. (2021). Evaluation of antioxidant, antibacterial and cytotoxicity activities of exopolysaccharide from Enterococcus strains isolated from traditional Iranian Kishk. *Journal of Food Measurement and Characterization*.

[97] Bubonja-Šonje M., Knežević S., Abram M. (2020). Challenges to antimicrobial susceptibility testing of plant-derived polyphenolic compounds. *Archives of Industrial Hygiene and Toxicology*.

[98] Al-Khikani F. (2020). Antimicrobial resistance profile among major bacterial pathogens in southern babil, Iraq. *Galician Medical Journal*.

[99] Bizri A. R., El-Fattah A. A., Bazaraa H. M. (2023). Antimicrobial resistance landscape and COVID-19 impact in Egypt, Iraq, Jordan, and Lebanon: a survey-based study and expert opinion. *PLoS One*.

[100] Azwanida N. (2015). A review on the extraction methods use in medicinal plants, principle, strength and limitation. *Medicinal & Aromatic Plants*.

